# Potential Use and Chemical Analysis of Some Natural Plant Extracts for Controlling *Listeria* spp. Growth *In Vitro* and in Food

**DOI:** 10.3390/foods13182915

**Published:** 2024-09-14

**Authors:** Abdul-Raouf Al-Mohammadi, Seham Abdel-Shafi, Ahmed H. Moustafa, Nehal Fouad, Gamal Enan, Rehab A. Ibrahim

**Affiliations:** 1 Department of Sciences, King Khalid Military Academy, Riyadh 11459, Saudi Arabia; almohammadi26@hotmail.com; 2 Department of Botany and Microbiology, Faculty of Science, Zagazig University, Zagazig 44519, Egypt; hegazyseham@yahoo.com (S.A.-S.); nehalfouad@yahoo.com (N.F.); gamalenan@ymail.com (G.E.); 3 Department of Chemistry, Faculty of Science, Zagazig University, Zagazig 44519, Egypt; ah_hu_mostafa@yahoo.com

**Keywords:** food-borne bacteria, *L. monocytogenes* LMG10470, *L. ivanovii* LMZ11352, essential oils, plant extracts, imipenem, GC-MS, IR spectra

## Abstract

*Listeria* are Gram-negative intracellular foodborne pathogens that can cause invasive infections with high mortality rates. In this work, the antibacterial activity of ten essential oils, infusion extracts, and decoction extracts of some medicinal plants was tested against *Listeria monocytogenes* and *listeria ivanovii* strains. The effects of different physical conditions including temperature, pH, sodium chloride, and some organic acids were studied. The results showed that the water extracts gave the maximum bacterial inhibition, while ethanolic extract was inactive against the tested *Listeria* spp. The antibiotic sensitivity of *L. monocytogenes* LMG10470 and *L. ivanovii* LMZ11352 was tested against five antibiotics including imipenem, levofloxacin, amikacin, ampicillin, and amoxicillin. Imipenem was the most effective antibiotic, resulting in inhibition zones of 40 mm and 31 mm for *L. monocytogenes* and *L. ivanovii*, respectively. When imipenem mixed with *Syzygium aromaticum* oil, *Salvia officinalis* oil, *Pimpinella anisum* infusion, and *Mentha piperita* infusion each, the water extract of *Moringa oleifera* leaves and seeds against LMG10470 and LMZ11352 resulted in broader antibacterial activity. The antimicrobial activity of both *Pimpinella anisum* and *Mentha piperita* plant extracts is related to a variety of bioactive compounds indicated by gas chromatography–mass spectrometry analysis of these two plant extracts. These two plant extracts seemed to contain many chemical compounds elucidated by gas chromatography–mass spectrometry (GC-MS) and infrared radiation spectra. These compounds could be classified into different chemical groups such as ethers, heterocyclic compounds, aromatic aldehydes, condensed heterocyclic compounds, ketones, alicyclic compounds, aromatics, esters, herbicides, saturated fatty acids, and unsaturated fatty acids. The use of these natural compounds seems to be a useful technological adjuvant for the control of *Listeria* spp. in foods.

## 1. Introduction

One of the most crucial problems facing the food sector is food safety. In actuality, food producers, consumers, and regulatory agencies are all concerned about pathogenic bacteria that cause foodborne illnesses [1]. The food business thus aspires to create food that is both safe and of the highest quality [2]. As a result, a portion of research efforts has always been focused on expanding our understanding of how to produce food that is safe and developing novel techniques to enhance that safety. Numerous sources, including soil, decomposing vegetation, silage, sewage, water, animal feed, fresh and processed foods, raw milk, cheese, abattoir waste, and asymptomatic human and animal carriers, have been linked to the isolation of *Listeria* species [3]. *Listeria* species are widely distributed and hence have several opportunities to infiltrate the food production and processing chain.

*Listeria monocytogenes* and *Listeria ivanovii* are Gram-positive intracellular foodborne pathogens that can cause invasive infections with high mortality rates, compared to many other foodborne pathogens [4]. Listeriosis is caused by the pathogenic bacteria *L. ivanovii* and *L. monocytogenes*. *L. ivanovii* is mostly an animal pathogen that sporadically causes sickness in people, whereas *L. monocytogenes* is an infection that can affect both humans and animals [5]. Outbreaks of listeriosis have frequently been linked to goods including dairy, eggs, meat, and fish [6]. Pregnancy-related listeriosis includes maternal, fetal, and neonatal infections. Neonatal listeriosis has a 20% case fatality rate and can result in meningitis or sepsis with serious aftereffects [7].

*L. monocytogenes* poses a challenge as a food-borne disease because of its high tolerance for salt chloride and ability to multiply at a comparatively low pH. For example, *L. monocytogenes* may develop at a pH of 7 at 25 °C in solutions containing 10% NaCl [8], continue to grow at 4 °C in 20% NaCI [9], and begin to grow at a pH of 4.39 at 30 °C [10]. This pathogen is also resistant to chilling temperatures. Determining the pathogen’s severity and controlling it requires an understanding of the effects of these physical factors. Rules pertaining to the handling and processing of foods at risk of *L. monocytogenes* contamination have been put in place because of the serious health danger that *L. monocytogenes* poses [8]. This is required because *L. monocytogenes* may be found in a variety of foods, including meat, fish, milk, cheese, fruits, and vegetables [11], and it is challenging to eliminate [12,13].

The essential elements of food safety laws impact work surfaces and packaging technologies in addition to personal hygiene. These days, disinfectants based on EDTA and hydrogen peroxide [14] ensure that surfaces are clean and effectively inhibit the growth of biofilms [15]. While washing with vinegar and water is advised for fruits and vegetables, pasteurization is the preferred process for milk [16,17]. *L. monocytogenes* can also be inhibited from growing by UV treatment and modified atmosphere packaging (MAP) [18,19,20]. Although chemical treatments (MAP and ozone) and washes (chlorine and organic acids) are generally effective in controlling this bacterium in food, natural solutions are receiving more attention due to the harmful effects of the widespread use of chemical preservatives in food [21,22].

As a result, research into creating novel, safe, and effective antibacterial substances has gained momentum. The use of natural antibacterial substances, such as extracts from medicinal plants, to preserve food is becoming more and more popular [10]. Plant-derived substances are abundant in phytochemicals, including phenolic acid, flavonoids, tannins, and lignin [11].

Recent years have seen a rise in the popularity of plant extracts, and efforts to identify the bioactive components of these extracts have gained traction for a variety of pharmaceutical and food processing uses. Numerous uses, such as the preservation of raw and processed food, pharmaceuticals, alternative treatments, and natural therapies, are based on the antibacterial properties of plant extracts [13].

The current manuscript aims to study (i). the effect of some physical factors on the growth of *L*. *monocytogenes* LMG10470 and *L. ivanovii* LMZ11352, (ii). the antibacterial activities of some essential oils, medicinal plant extracts, and antibiotics against *L. monocytogenes* LMG10470 and *L. ivanovii* LMZ11352, separately and in combination, and (iii). the determination of bioactive compounds of both *Pimpinella anisum* and *Mentha piperita* plant extracts by means of available instrumental analysis such as IR spectroscopy and GC-MS analysis.

## 2. Materials and Methods

### 2.1. Microbial Test Strains

Stock bacterial cultures of *Listeria monocytogenes* LMG10470 and *L. ivanovii* LMZ11352 were kept at −20 °C in glass beads and subcultured into brain heart infusion broth (BHI broth, Oxoid, Basingstoke, Hampshire, UK).

### 2.2. Essential Oils and Medicinal Plants

Essentials oils: Essential oils of the following herbs were purchased from El-Hawag Factory, Badr city, Egypt: clove (*Syzygium aromaticum*), chamomile (*Martricaria chamomilla*), rosemary (*Rosemarinus officinalis*), mint (*Mentha piperita*), black cumin (*Nigella sativa*), anise (*Pimpinella anisum*), thyme (*Thymus vulgaris*), ginger (*Zingiber officinalis*), sage (*Salvia officinalis*), and cinnamon (*Cinnamum zeylanieum*). All these essential oils are reagent grade.

Medicinal plants: The following medicinal plants were purchased from the local market in Sharkia Governorate (80 km north of Cairo): clove (*Syzygium aromaticum*), chamomile (*Martricaria chamomilla*), rosemary (*Rosemarinus officinalis*), mint (*Mentha piperita*), black cumin (*Nigella sativa*), anise (*Pimpinella anisum*), thyme (*Thymus vulgaris*), ginger (*Zingiber officinalis*), sage (*Salvia officinalis*), and cinnamon (*Cinnamum zeylanieum*).

### 2.3. Preparation of the Moringa Oleifera Leaf (MLE) and Seed (MSE) Extracts

In the Department of Botany and Microbiology, Faculty of Science, Zagazig University, Egypt, the plant taxonomist Prof. Dr. Hussein Abdel-Basset identified the plant *M. oleifera*. After gathering the seeds and leaves of *M. oleifera*, the leaves were thoroughly cleaned to remove any extraneous material, carefully washed, and dried in a hot air oven (Alexandria Co., Alexandria, Egypt) at 40 °C for 24 h. Using an airtight plastic container (Alexandria Co., Alexandria, Egypt), the seeds were sealed until needed. They were then dried and pounded into a powder using a sterile, clean mortar and pestle (Moulinex, Cairo, Egypt). Additionally, methanolic and ethanolic extracts were prepared by homogenizing 10 g of MSE or MLE with 100 mL of ethanol or methanol for 40 min [23]. The extracts were then left in an oven (Alexandria Co., Alexandria, Egypt) set to 60 °C for an overnight period to evaporate the solvents. The two types of extracts—leaf (MLE) and seed (MSE)—were homogenized using sterile water and filtered (0.45 milipore Bilters, Amicon, Mumbai, India) to ensure sterilization. Antimicrobial activity tests were conducted after stock preparation of MSE (200 μg/mL) was made and kept in Eppendorf tubes (Gomhuria Co., Zagazig, Egypt) at 5 °C [24].

### 2.4. Effect of Different Physical Factors on Listeria spp.

#### 2.4.1. Effect of Different Temperatures on *L. monocytogenes* and *L. ivanovii* Growth

Cell suspensions at 0.1 optical density and a wave length of 600 from *L. monocytogenes* and *L. ivanovii* were inoculated into BHI broths and incubated at 40 °C, 45 °C, 50 °C, 55 °C, 60 °C, 65 °C, and 70 °C for 24 h. Growth was tested by measuring turbidity at O.D_600_ (wave length).

#### 2.4.2. Effect of Different pH on *L. monocytogenes* and *L. ivanovii* Growth

*L. monocytogenes* and *L. ivanovii* strains were inoculated in nutrient broth adjusted at different pH values (2, 4, 6, 8, 10, and 12) by using a pH meter (Denver Instruments, Bohemia, NY, USA), and BHI was supplemented with appropriate combinations of sodium lactate + lactic acid or sodium acetate + acetic acid to achieve pH levels of 12.0, 11.0, 10.0, 9.0, 8.0, 7.0, 6.0, 5.0, and 4.0 in combination with concentrations of 0.1, 0.5, 1.0, and 2.0 M. These concentrations are roughly equivalent to 0.9, 4.5, 9.0, and 18.0% (wt/vol) for lactic acid and 0.6, 3.0, 6.0, and 12.0% for acetic acid, calculated on the basis of the acid and incubated for 24 h at 37 °C. Then, growth was determined by measuring cell turbidity at O.D_600_.

#### 2.4.3. Effect of Sodium Chloride Concentrations on *L. monocytogenes* and *L. ivanovii* Growth

Different concentrations of sodium chloride in nutrient broth media (5, 10, 15, 20, 25, and 30%) were made. *L. monocytogenes* and *L. ivanovii* strains at 0.1 OD were inoculated into these concentrations and incubated for 24 h at 37 °C; then, the growth was measured with cell turbidity at O.D600.

#### 2.4.4. Effect of Some Organic Acids on *L. monocytogenes* and *L. ivanovii* Growth

This was studied by using 3 different organic acids (oxalic acid, citric acid, and salicylic acid) with different concentrations in nutrient broth media (2, 4, 6, 8, 10, 12, and 14%). *L. monocytogenes* and *L. ivanovii* strains were inoculated into these concentrations and incubated for 24 h at 37 °C; then, growth was measured at OD 600.

### 2.5. Preparation of Infusion and Decoction Extracts

Infusion extracts were prepared by adding 10 g of the tested medicinal plants into 100 mL of distilled water and left for 24 h at room temperature with occasional shaking and filtrations to obtain clear infusion; then they were sterilized through a sterile microfilter (Millex-GV filter, 0.45 µm pore size, Millipore, Burlington, MA, USA). The prepared extraction was collected in sterilized glass bottles and stored in a refrigerator at 4 °C until used.

An aqueous decoction was prepared by boiling 10 g of the tested medicinal plants in 100 mL distilled water and left to infuse for 20 min. The decoction was then filtered through a sterile microfilter (Millex-GV filter, 0.45 µm pore size, Millipore, Burlington, MA, USA). The preparation was allowed to cool and was then collected in sterilized glass bottles and stored in a refrigerator at 4 °C until used [25].

### 2.6. Antibacterial Bioassays of Essential Oils and Medicinal Plant Extracts

Agar plates for brain heart infusion (BHI agar, Oxoid) were produced, and 10^6^ CFU/mL bacterial strains adjusted at O.D600 were added. Sterile glass rods were used to distribute the microbial inocula onto the agar plates in an absolutely aseptic manner. A sterile cork borer was used to create wells with a diameter of 10 mm. Next, 100 µL of sterile plant extracts were transferred into the agar plate wells that had been infected with the strains that had been tested. Prior to being treated, the agar plates were incubated at 35 °C for 48 h, and the plates were initially kept at 4 °C for 2 h to allow the prepared infusion to undergo pre-diffusion into the agar. Then, inhibition zone diameters were determined in accordance with the guidelines provided by the European Committee on Antimicrobial Susceptibility Testing (EU-CAST) and the Clinical Laboratory and Standard Institute (CLSI). The same steps were taken for the essential oils.

### 2.7. Determination of Minimum Inhibitory Concentration (MIC)

In accordance with the 2019 criteria published by the Clinical Laboratory Standards Institute (CLSI), the MIC values of the extracts were ascertained using the broth microdilution technique in 96-well microplates against all bacteria [25]. A total of 95 μL of sterile TSB were used to distribute 5 μL of each strain into each well of a sterile 96-well plate after the strain had been diluted overnight to a final concentration of 106 cfu/mL. After that, concentrations ranging from 512 to 0.125 μL/mL were obtained by adding 100 μL of extracted successive dilutions. Bacteria in TSB without extracts were present in the negative control wells. After mixing the plates for 20 s at 300 rpm on a plate shaker, they were incubated for 24 h at 37 °C. The lowest extracts were designated as the MIC value.

### 2.8. Quantitative Inhibition of Pathogenic Bacteria by Different Plant Extracts

A series of 250 mL Erlenmeyer flasks, each containing 100 mL aliquots of BHI broth (Oxoid), were sterilized by autoclaving at 120 °C for 15 min. After cooling, they were separately inoculated with 100 µL of log phase bacterial suspension, treated by 10 µL of the tested extract, and were then incubated in an incubator (New Brunswick Scien. Co., North Brunswick, NJ, USA) at 37 °C for 36 hr. Growth was then determined via the turbidity method with an OD_600_ spectrophotometer (Benchmark Accuris SmartReader 96 Plate Reader, [26].

### 2.9. Antibiotic Sensitivity Test and Antibacterial Activity of Natural Extract–Antibiotic Combinations by Disc Diffusion Assay

Ready antibiotic discs of imipenem (10 μg), levofloxacin (5 μg), amikacin (30 μg), ampicillin/sulbactam (10/10 μg), and amoxicillin (20/10 μg) were laid with appropriate distances separating them from each other on the surface of BHIB media seeded with all the tested bacteria. The plates were incubated at 37 °C for 24 h, and diameters of inhibition zones (mm) were measured as above. Results of an antibiotic sensitivity test were taken according to the instructions of CLSI (2008) [27].

The antibiotic imipenem that inhibited the *L.monocytogenes* and *L. ivanovii* strain was mixed with MIC values of the tested natural extract. Sterile filter paper discs were impregnated by these combinations and assayed for their antistaphylococcal activity as described above. In addition, different concentrations of either imipenem or natural extract were tested individually for their antilisterial activity. Different mixtures of *Syzygium aromaticum* oil–imipenem, *Salvia officinalis* oil–imipenem, *Pimpinella anisum* infusion (10%)–imipenem, *Mentha piperita* infusion (10%)–imipenem, the aqueous extract of *Moringa oleifera* leaves–imipenem, and the aqueous extract of *Moringa oleifera* seeds–imipenem were prepared. Paper discs of a 6 mm diameter were soaked with previous natural extract–antibiotic combinations, and the experiment was carried out as described previously [28].

### 2.10. Instrumental Analysis of Mentha and Ansium Infusion Extracts

By using GC-MS analysis, the infusions’ chemical makeup was determined. Helium was used as the carrier gas, and the apparatus was linked to an AT WAX 30 m × 0.32 mm × 1 µm capillary column (GS/MS QP 2010 Plus, Shimadzu, Kyoto, Japan). The flow rate of the helium was 1 mL/min. The following actions were taken, in brief: (i) the temperature program started at 40 °C and increased by 5 °C/min to 210 °C (for 5 min), the injector temperature reached 250 °C, and the ion source reached 220 °C; (ii) the injection volume was 1 µL at a split ratio of 1:50; the samples were diluted by 1:10 (*v*/*v*); and the solvent was n-hexane. Using the NIST 02 and Wiley 275 library spectra databases, the volatile molecules 2022, 27, 6106 16 of 21 were compared and identified. Using a Farrier transform infrared (FTIR) spectrometer (Bruker Optik GmbH, Ettlingen, Germany), the obtained mentha and ansium extracts’ infrared spectra were measured in accordance with the methodology described by earlier studies [23] to ascertain the presence of different functional groups in the extracts. To obtain the pellets needed for FTIR analysis, 100 mg of dry potassium bromide powder (KBr) and 1 mg of freeze-dried ansium or mentha powder were ground together and then pressed into a mold. At a resolution of 4 cm^−1^, the FT-IR spectra were captured in the 3500–500 cm^−1^ range. The spectroscopic software program OPUS/IR NT4.0 (Bruker Optik GmbH) was utilized to process the obtained data on the FTIR apparatus. By comparing the components’ retention durations and mass spectra to those of the WILEY 09 and NIST 11 mass spectral databases, the components were identified.

### 2.11. Statistical Analysis

Statistical analysis was performed on SPSS v.17.0 statistics software. Statistical differences and significance were assessed by a one-way ANOVA test and Wilcoxon signed ranks test, as appropriate, to evaluate the antibacterial inhibition according to the type of strains and the *Listeria* spp. A *p*. value < 0.05 was considered significant.

## 3. Results

**Figure 1:**
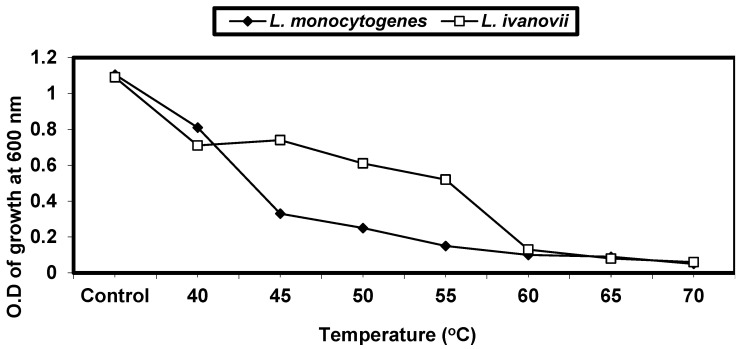
Thermal death point of *L. monocytogenes* LMG10470 and *L. ivanovii* LMZ11352 after 15 min of exposure to different temperatures different temperature exposure.

*L. monocytogenes* and *L. ivanovii* were incubated at different temperatures (40, 45, 50, 55, 60, 65 and 70 °C) for 24 h. Then, the growth was measured, with optical density at 600 nm. The control was bacteria without exposure to any temperature (the bacteria were inoculated, then incubated directly at 37 °C for 24 h). The results in (Figure 1) indicate that *L. monocytogenes* and *L. ivanovii* have the ability to grow at incubation temperatures from 40 to 55 °C. This indicated the ability of this genus to grow in severe conditions. The thermal death point was in the range of 55–60 °C.

**Figure 2:**
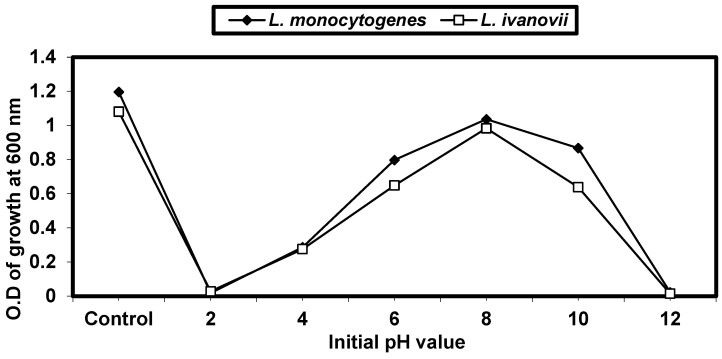
Effect of different pH values on *L. monocytogenes* LMG10470 and *L. ivanovii* LMZ11352 growth.

*L. monocytogenes* and *L. ivanovii* were exposed to different pH values (2, 4, 6, 8, 10, and 12), then incubated at 37 °C for 24 h. The growth was measured by optical density at 600 nm. The control was bacteria growing at a pH of 7.0. The growth of *L. monocytogenes* and *L. ivanovii* decreased at both high acidity (pH 2.0) and high alkalinity (pH 12.0). The results in (Figure 2) show that *Listeria* spp. almost grew well at a pH range of 4–10.0.

**Figure 3:**
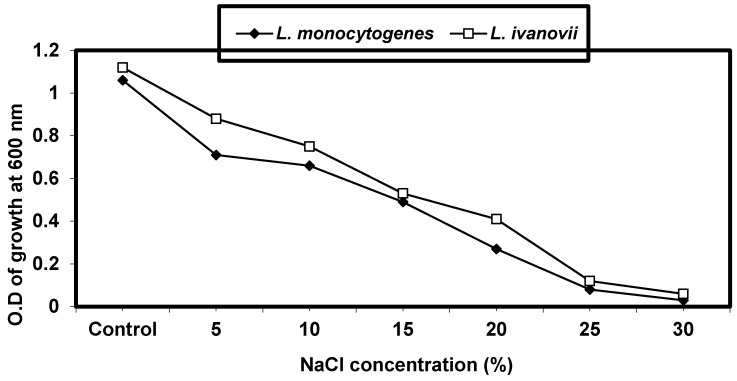
Effect of different NaCl concentrations on *L. monocytogenes* LMG10470 and *L. ivanovii* LMZ11352 growth.

*L. monocytogenes* and *L. ivanovii* were grown in media supplemented with different NaCl concentrations (5, 10, 15, 20, 25, and 30%) incubated at 37 °C for 24 h, and then the growth was measured by optical density at 600 nm. The results in (Figure 3) show that *L. monocytogenes* and *L. ivanovii* grew well at NaCl concentrations of about 5–20% and decreased at 25% NaCl concentrations.

**Figure 4:**
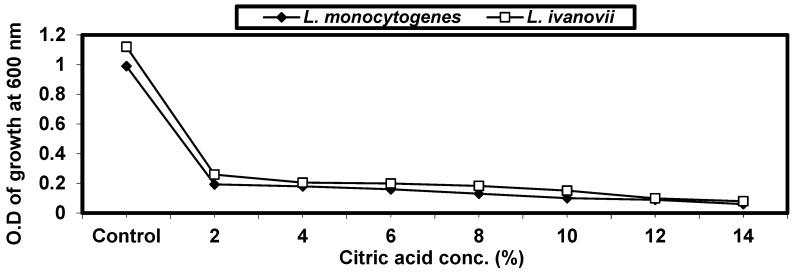
Effect of citric acid concentration on *L. monocytogenes* LMG10470 and *L. ivanovii* LMZ11352 growth.

Different concentrations of citric acid (2, 4, 6, 8, 10, 12, and 14%) were made in test tubes containing BHI broth; the tubes were then inoculated with both experimental organisms and incubated at 37 °C for 24 h. The results are given in (Figure 4). It was shown that the growth of both tested strains decreased by increasing citric acid concentrations up to 14%. At this concentration, the growth of *listeria* cells are rather low or prevented.

**Figure 5:**
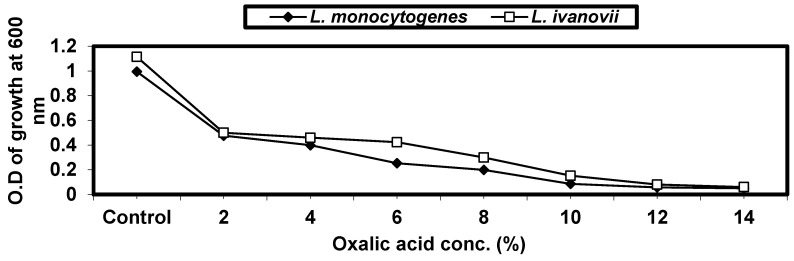
Effect of oxalic acid concentrations on *L. monocytogenes* LMG10470 and *L. ivanovii* LMZ11352 growth.

Different concentrations of oxalic acid (2, 4, 6, 8, 10, 12, and 14%) were tested against *L. monocytogenes* and *L. ivanovii* growth in BHI broth throughout incubation at 37 °C for 24 h; growth was monitored at O.D 600 nm. The data in (Figure 5) indicate that the growth decreased by increasing concentrations of oxalic acid up to 12%.

**Figure 6:**
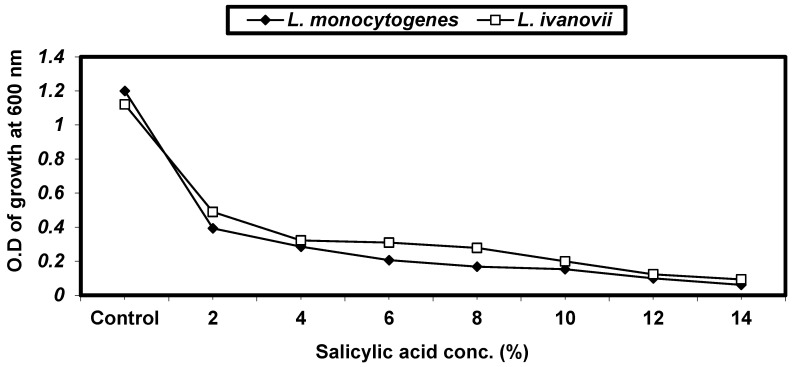
Effect of salicylic acid concentrations on *L. monocytogenes* LMG10470 and *L. ivanovii* LMZ11352 growth.

To study the effect of salicylic acid concentrations on *L. monocytogenes* and *L. ivanovii* growth, *Listeria* spp. were exposed to different concentrations of salicylic acid (2, 4, 6, 8, 10, 12, and 14%) in BHI broth media throughout incubation at 37 °C for 24 h. The growth of *listeria* cells in the treated samples was lower than that obtained in control (untreated sample). *listeria* cells grew at ≤10% salicylic acid. Higher concentrations <10% salicylic acid almost prevented the growth of cells (Figure 6).

Different essential oils were tested for their antibacterial action against *Listeria* spp. using disc assay and agar well diffusion methods (Table 1). It was shown that *Syzygium aromaticum* and *Salvia officinalis* oils (100 µL) had the ability to inhibit *L. monocytogenes* and *L. ivanovii* growth. However, other oils showed no antibacterial activity (Table 1).

The ten tested medicinal plants (100 µL) had potential antibacterial activity against both *L. monocytogenes* and *L. ivanovii* (Table 2). The aqueous infusion of *Pimpinella anisum* exhibited maximum activity against *L. monocytogenes*, with a 32 mm mean diameter of inhibition zone compared to other medicinal plants. Also, the infusion extract of *Mentha piperita* showed the highest antibacterial activity against *L. ivanovii* with an inhibition zone of about 29 mm by the disc diffusion method.

The results given in (Table 3) show the antibacterial activity of decoction extracts of ten medicinal plants. *Syzygium aromaticum*, *Rosemarinus officinalis*, and *Cinnamum zeylanieum* had weak inhibitory action against *L. monocytogenes* and *L. ivanovii*, but the other decoction extracts tested showed no antibacterial activity against the *Listeria* spp. tested.

**Figure 7:**
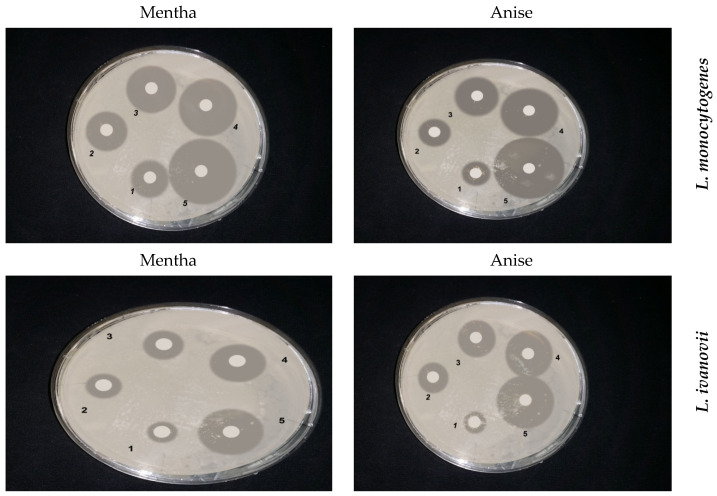
Antibacterial activity of different concentrations of infusion extracts of *Mentha piperita* and *Pimpinella anisum* against *L. monocytogenes* LMG10470 and *L. ivanovii* LMZ11352 by the disc assay method. The numbers 1, 2, 3, 4, and 5 show inhibition zone diameters of 10%, 25%, 50%, 75%, and 100%.

From the previous results, we observed that the infusion extracts that had the largest inhibition zones against *Listeria* ssp. were *Pimpinella anisum* and *Mentha piperita*. Different concentrations of these medicinal plant extracts were made and bioassayed against *Listeria* spp. The results are given in (Figure 7). It was shown that by increasing the concentration of medicinal plant extracts, the antibacterial activity against *L. monocytogenes* and *L. ivanovii* increased.

MIC was performed using both infusion extracts and decoction extracts. The results are given in (Table 4). The MIC values of the infusion extracts of both *P. anisum* and *M. piperita* were 0.62 g/100 mL and 2.5 g/100 mL using *L. monocytogenes*, and they were 2.5 g/100 mL and 2.5 g/100 mL using *L. ivanovii*; also, decoction extracts of *C. zeylanieum* and *S. aromaticum* extracts were 5 g/100 mL and 5 g/100 mL using *L. monocytogenes*, and they were 2.5 g/100 mL and 5 g/100 mL using *L. ivanovii*. 

**Figure 8:**
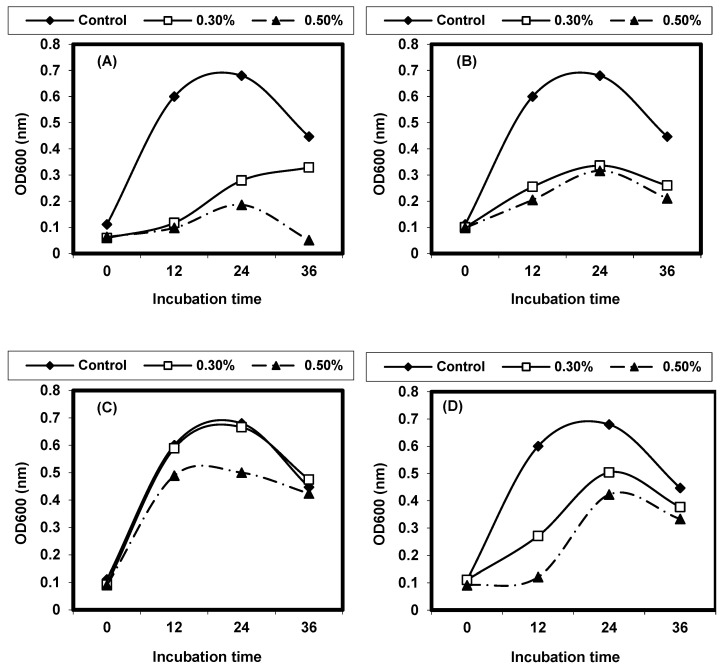

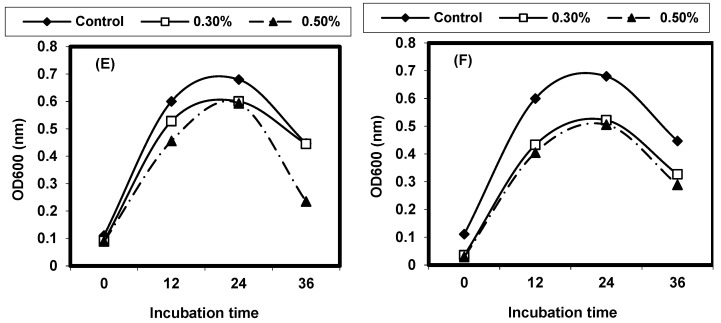
Growth curves of (**A**) Pimpinella anisum, (**B**) Mentha piperita, (**C**) Zingiber officinalis, (**D**) Rosemarinus officinalis, (**E**) Salvia officinalis, and (**F**) Martricaria chamomilla against L. monocytogenes LMG10470 in nutrient broth incubated at 37 °C for 24 h.

**Figure 9:**
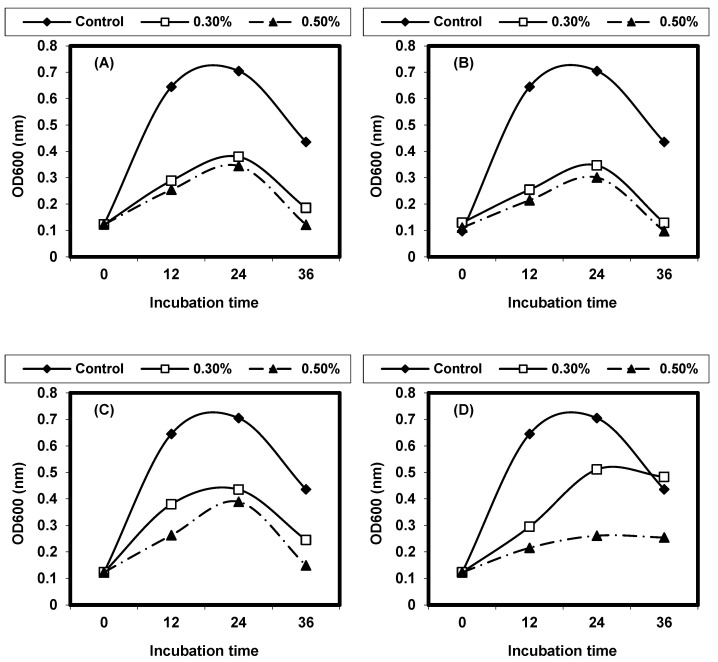

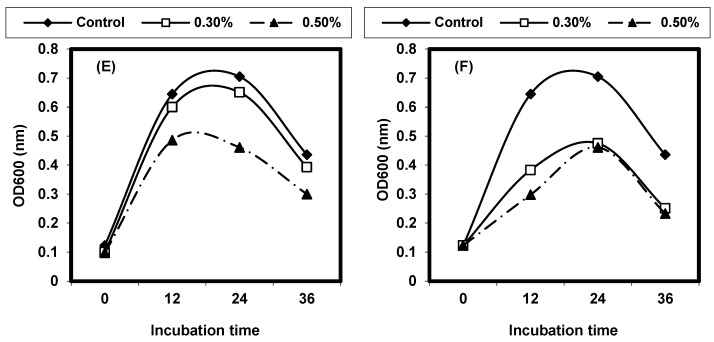
Growth curves of (**A**) *Pimpinella anisum*, (**B**) *Mentha piperita*, (**C**) *Zingiber officinalis*, (**D**) *Rosemarinus officinalis*, (**E**) *Salvia officinalis*, and (**F**) *Martricaria chamomilla* against *L. ivanovii* LMZ11352 in nutrient broth incubated at 37 °C for 24 h.

The results in (Figure 8 and Figure 9) quantitatively evaluate the antibacterial activities of infusion extracts with two concentrations (0.3% and 0.5%) of the tested medicinal plants in liquid media, and measurement of the growth at OD 600 is monitored. The growth curve of *L. ivanovii* was followed during 36 h at 37 °C as influenced by the presence of medicinal plants. A distinctive inhibition of *L. ivanovii* growth was detected by both *P. anisum* and *M. piperita* infusion extracts. Also, the infusion extract of *Z. officinalis* inhibited *listeria* growth; about 0.3 OD at 600 nm was observed between the treated sample and control. The growth of *listeria* cells treated by infusion extracts of other medicinal plants, showed comparable growth to controls but was rather lower than that obtained in the control experiments.

**Figure 10:**
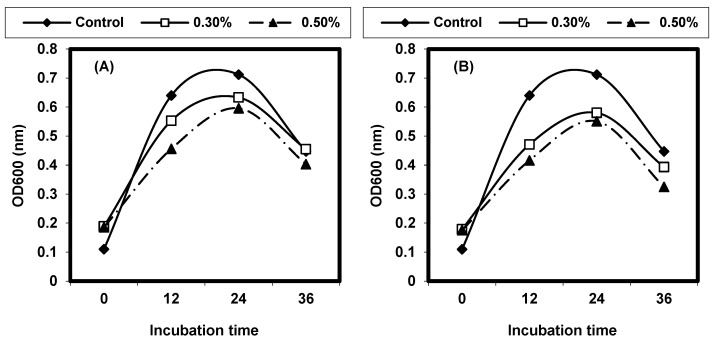

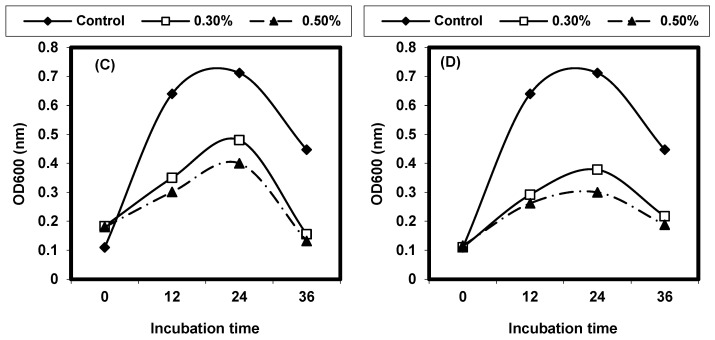
Quantitative inhibition of decoction extract of the test medicinal plants against *L. monocytogenes* LMG10470. (**A**) *Pimpinella anisum*, (**B**) *Rosemarinus officinalis*, (**C**) *Cinnamum zeylanieum*, and (**D**) *Syzygium aromaticum*.

The decoction extracts of some medicinal plants were assayed for the inhibition of L. monocytogenes. The results are given in (Figure 10). The decoction extracts of S. aromaticum and C. zeylanieum inhibited the growth of L. monocytogenes, but other decoction extracts of the other medicinal plants tested showed no effect.

**Figure 11:**
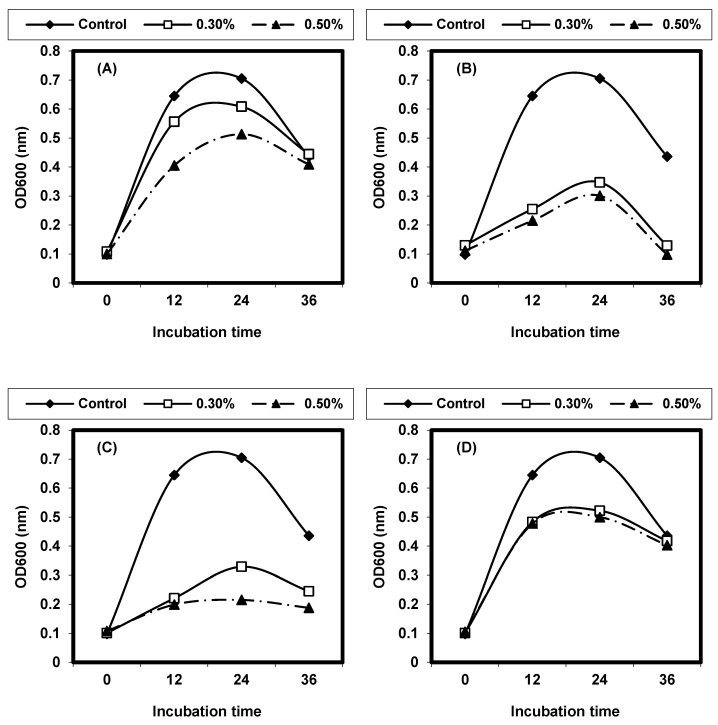
Quantitative inhibition of decoction extract of test medicinal plants against *L. ivanovii* LMZ11352. (**A**) *Pimpinella anisum*, (**B**) *Rosemarinus officinalis*, (**C**) *Cinnamum zeylanieum*, and (**D**) *Syzygium aromaticum*.

*L. ivanovii* was exposed to decoction of the tested medicinal plants. The growth of *L. ivanovii* was monitored by the measurement of turbidity at OD 600 nm. *C. zeylanieum* and *R. officinalis* had the best inhibition. A concentration of 0.5% of medicinal plants resulted in more inhibition effects than the 0.3% concentration against *L. ivanovii* (Figure 11).

**Figure 12:**
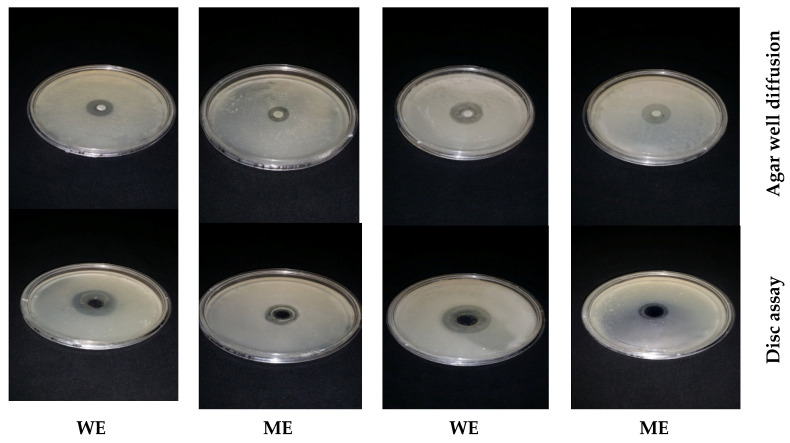
Antibacterial activity of *Moringa oleifera* extracts (leaves) against *L. monocytogenes* LMG10470 and *L. ivanovii* LMZ11352 using disc assay and agar well diffusion methods. WE: water extract of leaves. ME: methanol extract of leaves.

**Figure 13:**
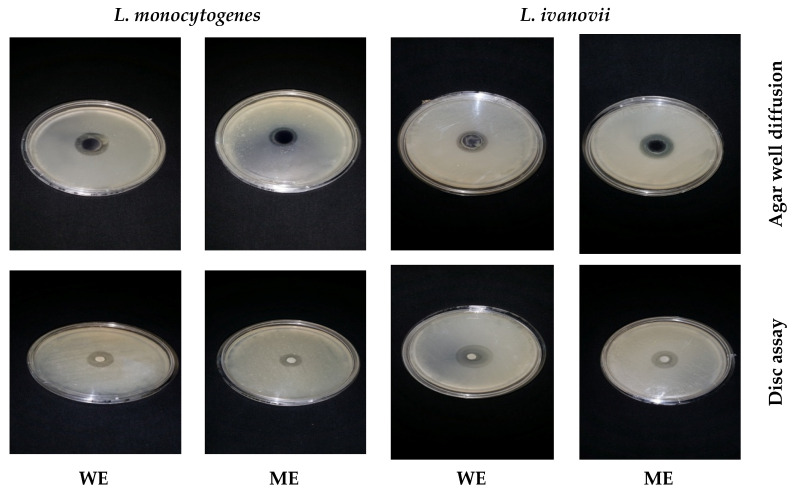
Antibacterial activity of *Moringa oleifera* extracts (Seeds) against *L. monocytogenes* LMG10470 and *L. ivanovii* LMZ11352 using disc assay and agar well diffusion methods. WE: water extract of seeds. ME: methanol extract of seeds.

Different solvent extracts (water, methanol, and ethanol) of the leaves and seeds of *Moringa oleifera* were used to inhibit *L. monocytogenes* and *L. ivanovii*. The water extracts and methanolic extracts of (leaves or seeds) inhibited distinctive *listeria* cells, while ethanolic extract was inactive against the tested *Listeria* spp. shown in (Figure 12 and Figure 13).

Five antibiotics (imipenem (10 µg), levofloxacin (5 µg), amikacin (30 µg), ampicillin (10/10 µg), and amoxicillin (20/10 µg)) were used for carrying out the antibiotic sensitivity test. The results in (Table 5) showed that the maximum inhibition zone was from imipenem, with inhibition zones of about 40 mm against *L. monocytogenes* and 31 mm against *L. ivanovii*. Levofloxacin showed minimum inhibitory activity against *L. ivanovii* (14 mm), while Amoxicillin had minimum inhibitory activity against *L. moncytogenes* (12 mm).

**Figure 14:**
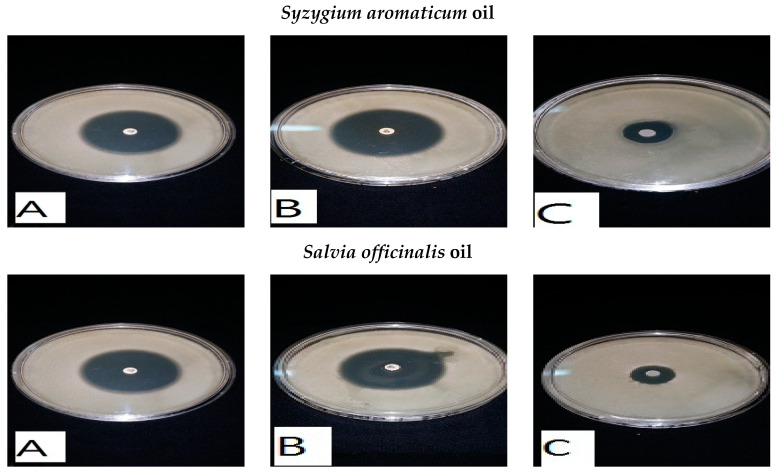

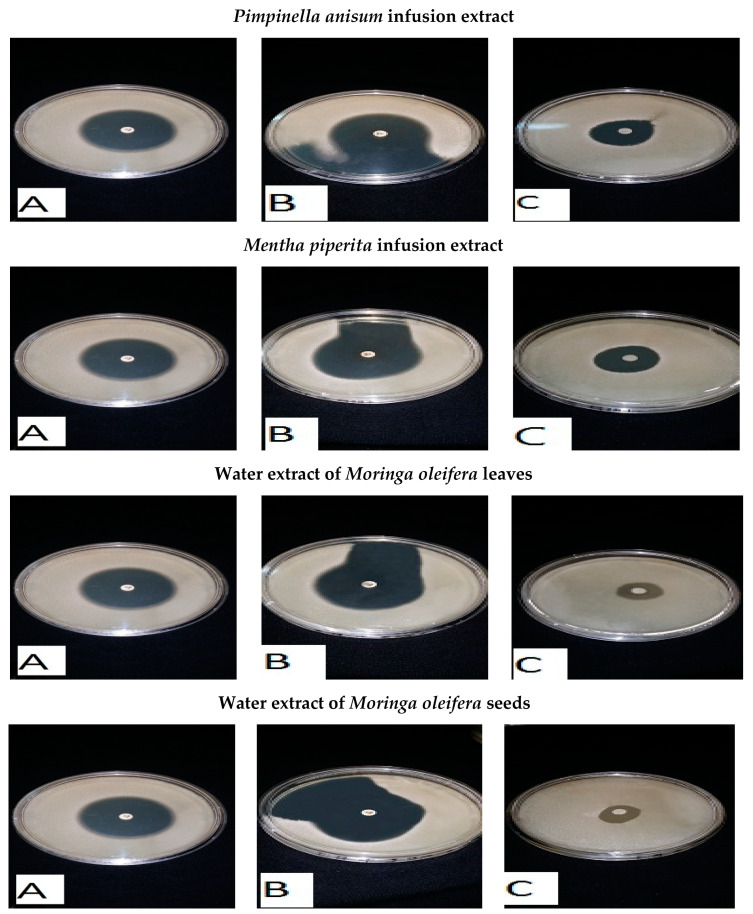
Antibacterial activity of mixed combinations of natural extracts and an antibiotic (imipenem) against *L. monocytogenes* by the disc assay method. (**A**) imipenem against *L. monocytogenes*. (**B**) (imipenem–natural extract) mixture combination against *L. monocytogenes*. (**C**) natural extract against *L. monocytogenes*.

**Figure 15:**
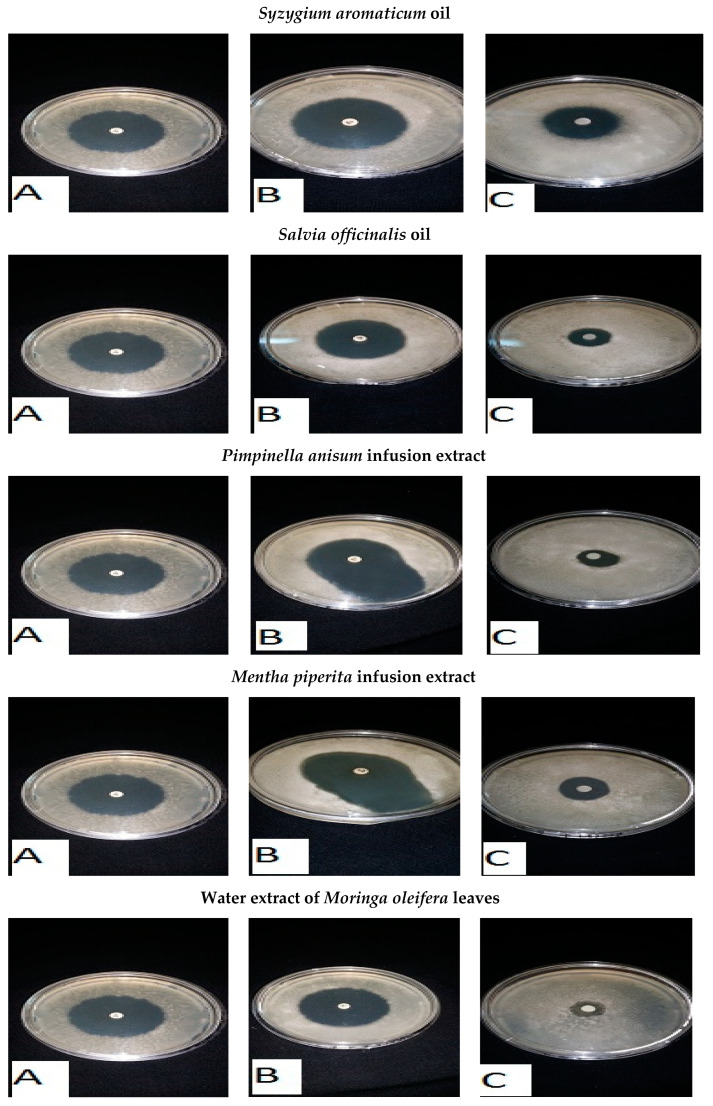

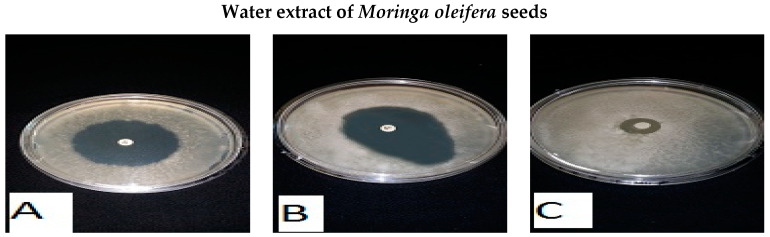
Antibacterial activity of mixed combinations of natural extracts and an antibiotic (imipenem) against *L. ivanovii* by the disc assay method. (**A**): imipenem against *L. ivanovii*. (**B**): (imipenem—natural extract) mixture combination against *L. ivanovii.* (**C**): natural extract against *L. ivanovii*.

The antibiotic with the best antibacterial activity was chosen in synergistic experiments with natural extracts that had antibacterial activity, and these included *S. aromaticum* oil, *S. officinalis* oil, *P. anisum* infusion extract, *M. piperita* infusion extract, and the water extract of *M. oleifera* leaves and *M. oleifera* seeds. The results are given in (Figure 14 and Figure 15). The combined effect of the imipenem–natural extract was greater than the antibacterial effect of the natural extract by only 3–8 times and was greater than the antibacterial effect produced by the antibiotic imipenem by almost 30%. This showed that mixed combinations of natural extracts with imipenem showed positive synergistic actions, which doubled the antibacterial activity against the *Listeria* spp. tested.

**Figure 16:**
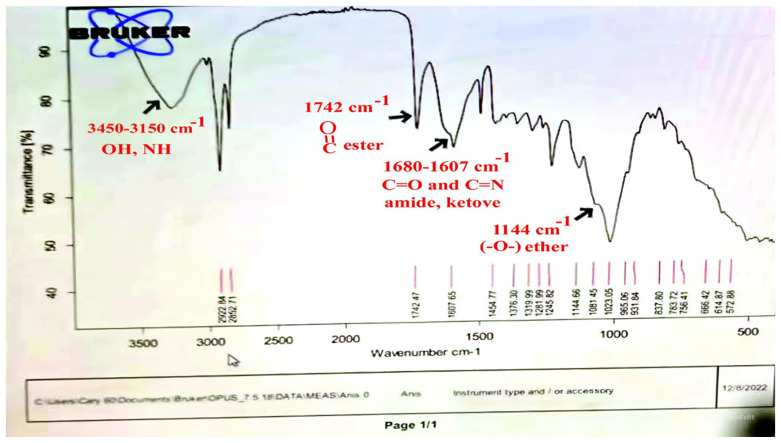
IR spectrum of *Pimpinella anisum.* Growth of *L. monocytogenes* LMG10470 (CFU/mL) in the presence of *L. delbreukii* subsp. *bulgaricus* Z55, *E. faecium* NM2, and *L. plantarum* LPS10 in vitro.

Both *Pimpinella anisum* and *Mentha piperita* were subjected to GC-MS analysis to detect their bioactive compounds. The results given in Table 6 and Table 7 show the compounds’ names and classes, in addition to molecular formula and molecular weight, for the chemical categories produced. The main compounds in the *Pimpinella anisum* are the ethers Estragole, Anethole, 1,2-Dimethoxy-Anethole, 1,2-Dimethoxy-4-(2-propenyl)- benzene, or Methyleugenol; 1,2-Dimethoxy-4-n-propylbenzene; the heterocyclic compound 5-Hydroxymethyl furfural; the aromatic aldehyde 4-Methoxybenzaldehyde; the condensed heterocycle 6-Methoxy-3-methyl-1-benzofuran; the ketone p-Methoxyphenyl-2-propanene; the alicyclic compounds longifolen (V4) and α-logipinene; the aromatic 1-(1,5-Dimethyl-4-hexenyl)-4-logipinene; the aromatics 1-(1,5-Dimethyl-4-hexenyl)-4-methylbenzene and B-Bisabolene; the esters 2-Methyl 4-methoxy-2-(1E)-1-propen-1-phenylbutanoate, Methyl(3,4-dimethoxy-phenyl)(hydroxy) acetate, and Bis(2-ethylhexyl)phthalate; the herbicide 3-Hydroxycarbofuran; the saturated fatty acid Hexadecanoic acid; the unsaturated fatty acid Cis-9,Cis-12-Octadecadiienoic acid; and the saturated fatty esters Methylhexadecan-oate). In addition, the IR spectrum (Figure 16) of the extracted ansium sample was obtained in KBr discs and showed the charaNH acid and amide groups, 1742 cm^−1^ C=O of ester, 1680–1607 cm^−1^ for 2 C=O amide, ketone, and C=N groups, in addition to band at 1144 cm^−1^ for the –O- ether groups.

**Figure 17:**
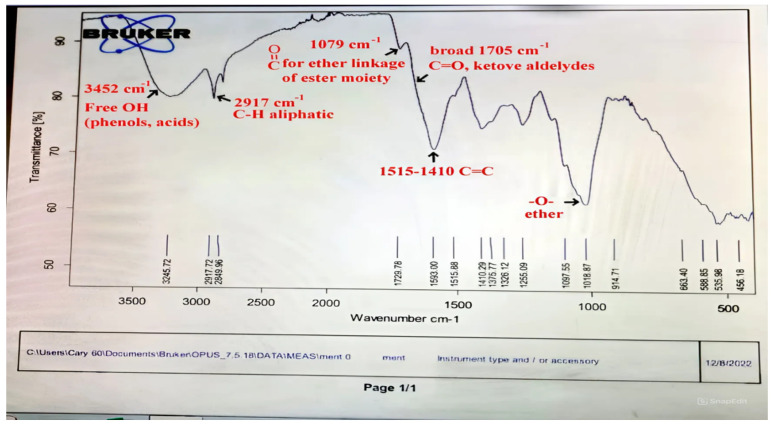
IR spectrum of *Mentha pipertia*.

The chemical composition of *Mentha pipertia* was detected using GC-MS analysis, and it showed the presence of the following bioactive compounds: alkenes: 2,6-Dimethyl-1,3,5,7-octatetraene; meophytadiene; alkaloides: Limonene, Cis-p-Menthan-3-one, Citronellal, (-)-Carvone; cyclicf ether: Cineole; heterocyclic: 2-Furylmethanol; ketone: 5-Methyl-2-(1-methyl-ethylidene) cyclo-hexanone; aldehyde: 4-(2,2-Dimethyl-6-methylenecyclohexyl)butanal; phenols: 5-Isopropyl-2-methylphenol, 3- Allyl-2-methoxyphenol; alicyclic compounds: α-Bourbonene, 2,6,10,10- Tetramethyl-bicyclo [7.2.0]undeca-1,6-diene; polynuclears: 1,2,4,5,6,8-Hexahydro-1-isopropyl-4,7-dimethyl naphthalene, Cis-Calamenene; saturated fatty ester: Methylpalmitate; saturated fatty acid: Hexadecanoic acid; unsat.fatty acid: Linolenic acid; and ester: Bis (2-ethylhexyl) phthalate. The IR spectrum (Figure 17) of Mentha in KBr (discs) gave the characteristic bands at υ cm^−1^ of a broad 3425 cm^−1^ for the free phenolic and acidic (OH) groups, 2917 cm^−1^ for stretching C-H aliphatic, 1729 cm^−1^ characteristic for the C=O of the ester group, 1705 cm^−1^ for the C=O of aldehydic and ketones, and 1515–1410 cm^−1^ for the C=C group, in addition to a band of 1097 cm^−1^ for the ether linkage of the ester moiety. All the bands characterized the functional groups in the extracted Mentha plants.

## 4. Discussion

One of the most significant pathogenic microbes is *Listeria monocytogenes*, which is the cause of listeriosis, an ailment mostly affecting the elderly, young children, pregnant women, and those with weakened immune systems [29]. In the ready-to-eat (RTE) meat and dairy product industries, this microorganism is a serious concern [30,31,32]. It can colonize and grow in raw materials and pre-processed products during processing and/or storage, putting consumers at risk and/or causing non-compliance with microbiological criteria for this pathogenic bacterium.

The emergence and dissemination of microorganisms resistant to antibiotics is a major global public health concern. Numerous bacteria have developed, and they maintain near-total resistance to almost all antimicrobial agents. In underdeveloped nations, resistance rates are higher [33]. To address the difficulties brought on by the rise in bacterial resistance, new preventive and therapeutic measures must be developed immediately [34].

*Listeria* species are remarkable in that they can multiply over a temperature range of −0.4 °C to 45 °C, with 37 °C being the ideal temperature [35]. They can also tolerate a wide range of pH values, from 4.6 to 9.5, salt concentrations up to 20%, and relatively low water activity (aW < 0.90) [36]. These bacteria’s developmental circumstances allow them to endure and proliferate in unfavorable environmental settings, which are frequently seen in food manufacturing facilities [37]. According to this perspective, an effort was made to regulate *L. monocytogenes* and *L. ivanovii* by utilizing a few physical parameters, such as pH, temperature, sodium chloride, and some organic acids. In this paper, the effects of various concentrations of these chemicals against the examined foodborne bacteria were investigated.

Numerous physical and chemical stress variables have been identified as impacting *L. monocutogenes* and *L. ivanovii* survival. It was possible to obtain an attenuated variant of *L. monocytogenes* LMG10470 and *L. ivanovii* LMZ11352 by researching the effects of physical and chemical factors on these deadly organisms. This study examined the impact of heat on Lactobacillus monocytogenes and Lactobacillus ivanovii. The findings indicated that the effects varied depending on the duration of exposure and the temperature employed. It showed that the temperature range of 40 to 60 °C is suitable for the growth of *L. monocytogenes* and *L. ivanovii*. This indicates that this genus is able to flourish in harsh environments [37]. Temperatures of 65 °C and above completely stopped the growth of *L. monocytogenes* and *L. ivanovii* after 15 min of exposure. Recent research [38,39] has examined *L. monocytogenes’* resilience to heat in a variety of food types and has found that heating food to an internal temperature of 70 °C for two minutes is enough to ensure the pathogen’s removal.

Additionally, *L. monocytogenes* was subjected to the following pH ranges: 2, 4, 6, 8, 10, and 12. *Listeria* species grew most at a pH of 7 (control) and pH 8. Low pH has been demonstrated to give L. monocytogenes greater resistance against other unfavorable environmental circumstances, in addition to enhancing its virulence [40]. By developing a variety of strategies, such as the metabolic production of intracellular acids through the deamination of amino acids and the fermentation of sugars [41], the induction of trans-porters and enzymes directly responsible for proton retention, and modifications to the cell surface [42], *L. monocytogenes* and *L. ivanovii* are quite resistant to alkaline pH. It has been proven that monovalent cation proton antiporters are essential to maintain a neutral cytoplasmic pH and, therefore, to allow for bacterial growth under alkaline conditions, such as growing in liquid media at a pH of 10, and this is in agreement with [43].

It was investigated how varying amounts of sodium chloride affected *L. monocytogenes* and *L. ivanovii*. In line with earlier research [36], this current investigation showed that *L. monocytogenes* and *L. ivanovii* can withstand unfavorable salt levels up to 20% and can even grow in a medium supplemented with 12% NaCl. Elevated concentrations of NaCl restrict the development of bacteria by reducing the amount of water in the surrounding medium, promoting plasmolysis, which in turn lowers intracellular turgor pressure and ultimately stops the growth of bacteria [44].

The effect of organic acids on the growth of both *L. monocytogenes* and *L. ivanovii* was studied. It was found that there is an opposite relation between pH degree and acidity. Organic acids result in a decrease in pH value; this may influence growth by acidifying the cell, which will consume a great amount of energy to maintain intracellular pH homeostasis. Other explanations have also been proposed, including membrane disruption, the interruption of metabolic reactions, and the accumulation of toxic anions; this supports latter findings in this respect [45]. Similar results were reported by [46], who compared the antilisterial effects of low equal molar concentrations (0.083 M) of acetic acid (0.5%*v*/*v*, pH 2.90) and citric acid (1.6% *v*/*v*, PH 2.05).

Ten essential oils, infusion extracts, and decoction extracts of several medicinal plants were investigated for their antibacterial activity against *L. monocytogenes* and *L. ivanovii* in this current study. According to recent research [47], clove essential oil may be useful in inhibiting *L. monocytogenes* and extending the shelf life of meat [48,49]. Clove and sage essential oils also show antibacterial activity against *L. ivanovii* and *L. monocytogenes*. Eugenol, a substance of the phenylpropanoid family that degrades bacterial cell walls and lyses them, is linked to clove’s antibacterial activity [50,51].

Essential oils can be utilized to extend the shelf life of food products since they are often released as secondary metabolites with antibacterial, antifungal, and antibiofilm qualities [52].

Tested against *L. monocytogenes*, the bactericidal properties of many plant aqueous extracts, including the anise seed fruit extract, and of their separated components were shown to be in agreement with earlier research [53]. *Mentha piperita* is administered topically as a liniment or massage oil and is taken orally as a tea, tincture, oil, or extract. Its potential as an estrogen, antiseptic, antipruritic, antispasmodic, anticatarrhal, antibacterial, rubefacient, stimulant, and antioxidant is regarded by herbalists as being on par with that of butylated hydroxy toluene (BHT), a synthetic antioxidant [54].

Plant extracts demonstrate inhibitory action against *listeria* in both decoction and infusion forms. Decoction had the highest content of flavonoids and total phenolic compounds, followed by infusion extracts, suggesting that phenolic chemicals, primarily flavonoids, may be responsible for antioxidant action. According to earlier research, infusion and decoction extracts exhibited more antibacterial activity than hydroalcoholic extracts [50]. This finding is consistent with our research.

Furthermore, it was shown that the concentration of plant extracts significantly impacted the development of *listeria*, with a 0.5% concentration of either infusion or decoction extracts being more efficient than a 0.3% concentration in inhibiting the proliferation of *listeria*-treated cells. This might be connected to the rise in phenolic component content, which is mostly connected to the plant extracts under study having an inhibitory effect. [53]

The varying degree of sensitivity of the bacterial strains may be due to the intrinsic tolerance of the bacterial cell and the nature and combinations of phytocompounds present in the extracts as confirmed by previous studies [55].

In accordance with the results of GC-MS analysis of anisum and mentha infusion extracts, the presence of eleven groups in anisum and twelve different chemical groups in mentha were indicated, which were reported to exert strong antibacterial activity by different mechanisms of action [56]. A recent review by Ramos de Silva, et al., 2021 discussed the importance of the studied plant extracts as a potent antimicrobial and antioxidant, as well as other biological activities in the context of their chemical profiling [57]

Cell membranes are made of ethers. Due to their antibacterial properties, they can be used to treat illnesses brought on by both Gram-positive and Gram-negative bacteria by preventing the normal growth of the cell membrane and by interfering with the integrity and permeability of the cell [58].

The ability of alicyclic compounds, such as longifolen (V4) and α-logipinene, to permeabilize membranes, including mitochondrial membranes, and to destroy the cellular integrity of bacteria and eukaryotic cells, resulting in necrosis and apoptosis, may account for their antibacterial activity [59].

Alkaloids are considered to be antimicrobial due to their ability to disrupt membranes, rapidly denaturize proteins, and induce nutrition loss from cells [60]. Cell lysis and metabolism are hampered as a result [61].

The same phenols that were clarified in this investigation were also shown in another study to cause antibacterial activity by gradually leaking intracellular components, such as K^+^. The initial indicator of membrane breakdown [62] also prevents vital nutrients from being taken up, which leads to cell death.

The esters and fatty acid esters that are presented here are often more hydrophobic and positively charged. This hydrophobicity facilitates electrostatic interactions with the bacterial cell components, which results in the production of completely de-energized dead cells, which ultimately results in the loss of cell viability. Additionally, they function as surfactants, inhibiting the growth of five foodborne pathogens: *B. cereus*, *B. subtilis*, *S. aureus*, *E. coli* O157: H7, and Salmonella typhi-murium. Moreover, they function as antibacterial food additives by preventing the formation of biofilms and bacterial growth [28].

The polyalkenes discussed here have an additional antibacterial effect because of the repulsive force that forms between the positively charged polymers and the negatively charged bacteria. This force inhibits the permeability of cells [63].

Analgesic, anthelmintic, antitubercular, plant growth regulator, antiviral, antifungal, and anticancer agents have all been associated with heterocyclic compounds [64]. Through their capacity to interact with the cells’ electronophores or nucleophiles, they are able to inhibit the synthesis of cell walls, proteins, DNA, and metabolic pathways, as well as interfere with the integrity of cell membranes, demonstrating their antibacterial activity [65].

This study elucidated how aromatic aldehydes exhibit high bactericidal activity when they associate with the outer layer of bacterial cells [66]. Specifically, these aldehydes were shown to interact with unprotonated amines on the cell surface, which in turn influence ions’ transport across the cell wall. Testing each compound’s antibacterial activity independently will be required.

Tests were conducted using three different solvent extracts (water, methanol, and ethanol) of *Moringa oleifera* leaves and seeds against *L. monocytogenes* and *L. ivanovii*. The highest levels of bacterial inhibition were obtained by water and methanolic extracts, but ethanolic extract showed no activity against the tested strains of *Listeria*. Numerous active ingredients found in *Moringa oleifera*, including tannins, alkaloids, flavonoids, saponins, and triterpenoids, which have strong anthelmintic and antibacterial effects, can help to explain the plant’s antimicrobial activity [26,67,68].

Imipenem had a stronger antibacterial impact on both *Listeria monocytogenes* and *Listeria ivanovii* in this current investigation, which examined the susceptibilities of both species to five antibiotics in accordance with CLSI [27]. Imipenem has an inhibitory zone measuring approximately 40 mm for *L. monocytogenes* and 31 mm for *L. ivanovii*. This could be explained by the fact that imipenem is a member of the carbapenem class, which is known for being the preferred antibiotic against susceptible strains of *Listeria* and other Gram-positive bacteria because of its ability to quickly kill bacteria by inhibiting the synthesis of their cell walls through the prevention of trans-peptidation, which is essential to maintain the structural integrity of the bacterial cell wall [69]. However, carbapenem resistance has spread globally over the past ten-to-fifteen years, and the frequency of illnesses caused by these resistant isolates has surged [70]. Therefore, it becomes necessary to utilize plant extracts to regulate these resistant bacteria.

A wider range of antibacterial activity was observed when imipenem was combined with the oils of *Syzygium aromaticum*, *Salvia officinalis*, *Pimpinella anisum*, *Mentha piperita*, water extract of *Moringa oleifera* leaves, and *Moringa oleifera* seeds against *L. monocytogenes* LMG10470 and *L. ivanovii* LMZ11352 [71,72,73]. Antibiotics and plant extracts may work synergistically because of chemical interactions, hydrogen bonding, and hydrophobic–hydrophobic interactions. The examination of bioactive chemicals found in plant extracts, including polar and non-polar chemical moieties, confirms this and explains the synergism seen between carbapenems and the studied plant infusion extracts [27]. Actually, further research will be required to examine this kind of synergism at the molecular and chemical levels.

## 5. Conclusions

This study showed the anti-*L. monocytogenes* and *L*. *ivanovii* activity of different extracts derived from plants and essential oils. Furthermore, the active chemical components of the most effective plant extracts, namely *Pimpinella anisum* and *Mentha piperita*, were determined by GC-MS. The use of these natural compounds seems to be a useful technological adjuvant for the control of *L. monocytogenes* and *L. ivanovii* instead of the chemical methods used. However, further studies are necessary to better define the potential of these types of natural products in the field of bio-preservation, exploiting the possibility of synergies between them and the exact mechanisms of action of these bio-preservatives to reduce the risk of contamination in the food industry from *L. monocytogenes*.

## Figures and Tables

**Table 1:** Antibacterial activity of essential oils against *L. monocytogenes* LMG10470 and *L. ivanovii* LMZ11352 using disc assay and agar well diffusion methods.

Common Name	Scientific Name	Inhibition Zone Diameter (mm)			
		*L. monocytogenes*		*L. ivanovii*	
		Disc	Wells	Disc	Wells
Clove	*Syzygium aromaticum*	14 ± 0.9	15 ± 1.0	13 ± 0.8	14 ± 0.9
Chamomile	*Martricaria chamomilla*	-	-	-	-
Rosemary	*Rosemarinus officinalis*	-	-	-	-
Mint	*Mentha piperita*	-	-	-	-
Black cumin	*Nigella sativa*	-	-	-	-
Anise	*Pimpinella anisum*	-	-	-	-
Thyme	*Thymus vulgaris*	-	-	-	-
Ginger	*Zingiber officinalis*	N	N	N	N
Sage	*Salvia officinalis*	10 ± 2	17 ± 3	3 ± 1.0	9 ± 1.5
Cinnamon	*Cinnamum zeylanieum*	-	-	-	-

(-): No inhibition zone. (N): Neglectable.

**Table 2:** Antibacterial activity of the infusion of some medicinal plants against *L. monocytogenes* LMG10470 and *L. ivanovii* LMZ11352 using a disc assay.

Common Name	Scientific Name	Inhibition Zone Diameter (mm)	
		*L. monocytogenes*	*L. ivanovii*
		Disc	Disc
Clove	*Syzygium aromaticum*	3 ± 0.2	2 ± 0.2
Chamomile	*Martricaria chamomilla*	3 ± 0.2	9 ± 0.5
Rosemary	*Rosemarinus officinalis*	14 ± 1.0	18 ± 2.0
Mint	*Mentha piperita*	32 ± 2.0	29 ± 2.0
Black cumin	*Nigella sativa*	13 ± 3.0	10 ± 1.0
Anise	*Pimpinella anisum*	32 ± 2.0	27 ± 2.0
Thyme	*Thymus vulgaris*	2 ± 0.2	20 ± 0.3
Ginger	*Zingiber officinalis*	25 ± 1.0	24 ± 4.0
Sage	*Salvia officinalis*	5 ± 0.3	29 ± 4.0
Cinnamon	*Cinnamum zeylanieum*	3 ± 0.3	4 ± 0.5

**Table 3:** Antibacterial activity of decoction extracts of tested medicinal plants against *L. monocytogenes* LMG10470 and *L. ivanovii* LMZ11352 using disc assay and agar well diffusion methods.

Decoction of Medicinal Plants	Inhibition Zone Diameter (mm)			
	*L. monocytogenes*		*L. ivanovii*	
	Disc	Wells	Disc	Wells
*Syzygium aromaticum*	8	9	5	7
*Martricaria chamomilla*	-	-	-	-
*Rosemarinus officinalis*	1	1	2	2
*Mentha piperita*	-	-	-	-
*Nigella sativa*	-	-	-	-
*Pimpinella anisum*	-	-	-	-
*Thymus vulgaris*	-	-	-	-
*Zingiber officinalis*	-	-	-	-
*Salvia officinalis*	-	-	-	-
*Cinnamum zeylanieum*	3	3	5	8

(-): No inhibition zone.

**Table 4:** Determination of the minimum inhibitory concentration (MIC) of infusion and decoction extracts of tested medicinal plants against *L. monocytogenes* LMG10470 and *L. ivanovii* LMZ11352.

Microorganisms	MIC (μg/mL) of Infusion Extracts		MIC (μg/mL) of Decoction Extracts	
	*Pimpinella anisum* (g/100 mL)	*Mentha piperita* (g/100 mL)	*Cinnamum zeylanieum* (g/100 mL)	*Syzygium aromaticum* (g/100 mL)
*L. monocytogenes*	0.62	2.5	5	5
*L. ivanovii*	2.5	2.5	2.5	5

**Table 5:** Antibiotic sensitivity of *L. monocytogenes* LMG10470 and *L. ivanovii* LMZ11352 according to Clinical and laboratory standards institute CLSI (2006).

Names of Antibiotics μg	Inhibition Zone Diameter (mm)	
	*L. Monocytogenes*	*L. ivanovii*
Imipenem (10 μg)	40 ± 3.0	31 ± 2.5
Levofloxacin (5 μg)	20 ± 2.5	14 ± 2.0
Amikacin (30 μg)	24 ± 1.0	20 ± 2.0
Ampicillin/sulbactam (10/10) μg	21 ± 2.5	27 ± 1.0
Amoxicillin (20/10) μg	12 ± 2.0	27 ± 2.0

**Table 6:** Putative identification of the chemical components from Pimpinella anisum when subjected to GC-MS (gas chromatography–mass spectrometry).

	Classification and Compound Name	Mol.wt and Mol. Formula	Parent Ion (M^+^)	Area	Base Peak (*m*/*z*) (100%)
	**Group A: Ethers**				
**1.**	Estragole	C_10_H_20_O (148.0)	148.0	0.71	77.00
**2.**	Anethole 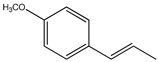	C_10_H_12_O (148.0)	148.0	43.64	77.00
**3**	**1,2-Dimethoxy-4-(2-propenyl)- benzene or Methyleugenol** 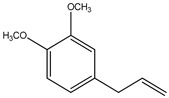	C_11_H_14_O_2_ (178.0)	178.0	0.60	91.00
**4**	**1,2-Dimethoxy-4-n-propylbenzene**	C_11_H_16_O_2_ (180.0)	180.0	0.35	151.0
	**Group B: Heterocyclic compound**				
**1.**	**5-Hydroxymethyl furfural** 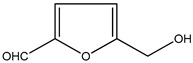	C_6_H_6_O_3_ (126.0)	126.0	1.21	97.00
	**Group C:** **Aromatic aldehydes**				
**1.**	4-Methoxybenzaldehyde 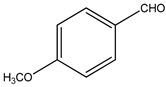	C_8_H_8_O_2_ (136.0)	136.0	0.99	77.00
	**Group D:** **Condensed Heterocyclic cpd**				
**1.**	**6-Methoxy-3-methyl-1-benzofuran**	C_10_H_10_ O_2_ (162.0)	162.0	0.68	147.0
	**Group E: ketone**				
**1.**	**p-Methoxyphenyl-2-propanene** 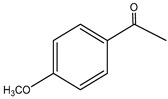	C_10_H_12_O_2_ (164.0)	164.0	1.49	121.0
	**Group F: Alicyclic compounds**				
**1.**	**ongifolene (V4)**	C_15_H_24_ (204.0)	204.0	10.15	105.0
**2.**	**α-logipinene**	C_15_H_24_ (204.0)	204.0	0.97	119.0
	**Group G: Aromatics**				
**1.**	**1-(1,5-Dimethyl-4-hexenyl)-4-methylbenzene** 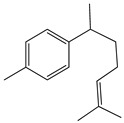	C_15_H_22_ (202.0)	202.0	1.29	119.0
**2.**	**B-Bisabolene**	C_15_H_24_ (204.0)	204.0	2.37	69.00
	**Group H: Esters**				
**1.**	**2-Methyl 4-methoxy-2-(1E)-1-propen-1-phenylbutanoate**	C_15_ H_20_O_3_ (248.0)	248.0	11.10	164.0
**2.**	**Methyl(3,4-dimethoxy-phenyl)(hydroxy) acetate** 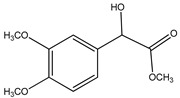	C_11_ H_14_O_5_ (226.0)	226.0	3.06	167.0
**3.**	**Bis(2-ethylhexyl)phthalate**	C_24_ H_38_O_4_ (390.0)	390.0	2.48	149.0
	**Group I: Herbicides**				
**1.**	**3-Hydroxycarbofuran**	C_12_H_15_NO_4_ (237.0)	237.0	7.62	137.0
	**Group J: saturated fatty acids**				
**1.**	Hexadecanoic acid CH_3_(CH_2_)_14_-COOH	C_16_H_32_O_2_ (256.0)	256.0	1.43	60.00
	**Group k: unsaturated fatty acid**				
**1.**	**Cis-9,Cis-12-Octadecadiienoic acid**	C_18_H_32_O_2_ (280.0)	280.0	3.20	67.00

**Table 7:** Putative identification of the chemical components from *Mentha pipertia* when subjected to GC-MS (gas chromatography–mass spectrometry).

	Classification and Compound Name	Mol.wt and Mol. Formula	Parent Ion (M^+^)	Area	Base Peak (*m*/*z*) (100%)
	**Group A: Alkenes**				
**1.**	2,6-Dimethyl-1,3,5,7-octatetraene	C_10_H_14_ (134.0)	134.0	2.50	91.00
**2.**	Meophytadiene	C_20_H_28_ (278.0)	278.0	6.99	68.00
	**Group B: Alkaloides**				
**1.**	**Limonene** 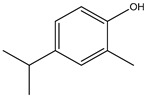	C_10_H_16_ (126.0)	136.0	0.90	68.00
**2.**	**Cis-p-menthan-3-one** 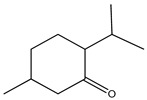	C_10_H_18_O (154.0)	154.0	1.46	41.00
**3.**	**Citronellal**	C_10_H_18_ O (154.0)	154.0	1.33	69&41
**4.**	**(-)-Carvone**	C_10_H_14_O (150.0)	150.0	56.39	82.00
	**Group C: Cyclic ether**				
**1.**	**Cineole**	C_10_H_18_O (154.0)	154.0	1.30	43.00
	**Group D:** **Heterocyclic cpd**				
**1.**	**2-Furylmethanol** 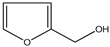	C_5_H_6_O_2_ (98.0)	98.0	1.07	39.00
	**Group E: ketone**				
**1.**	**5-Methyl-2-(1-methyl-ethylidene) cyclo-hexanone**	C_10_H_16_O (152.0)	152.0	1.57	81.00
	**Group F: Aldehydes**				
**1.**	**4-(2,2-Dimethyl-6-methylenecyclohexyl)butanal**	C_13_H_22_O (194.0)	194.0	0.73	69.00
	**Group F: Phenols**				
**1.**	**5-Isopropyl-2-methylphenol** 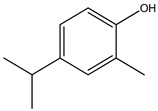	C_10_H_14_O (150.0)	150.0	0.77	135.0
**2.**	**3- Allyl-2-methoxyphenol** 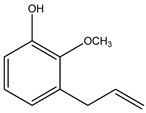	C_10_H_12_O_2_ (164.0)	164.0	0.91	91.00
	**Group F: Alicyclic compounds**				
**1.**	**α-Bourbonene**	C_15_H_24_ (204.0)	204.0	0.94	81.00
**2.**	**2,6,10,10- Tetramethyl-bicyclo [7.2.0]undeca-1,6-diene**	C_15_H_24_ (204.0)	204.0	1.71	41.00
	**Group G:Polynuclears**				
**1.**	**1,2,4,5,6,8-Hexahydro-1-isopropyl-4,7-dimethyl naphthalene**	C_15_H_24_ (204.0)	204.0	0.98	105.0
**2.**	**Cis-calamenene** 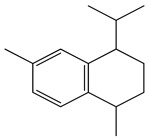	C_15_H_22_ (202.0)	202.0	1.72	159.0
	**Group H: saturated fatty ester**				
**1.**	**Methylpalmitate**	C_17_ H_34_O_2_ (270.0)	270.0	0.65	74.00
	**Group I: Saturated fatty acid**				
**1.**	**Hexadecanoic acid**	C_16_ H_32_O_2_ (256.0)	256. 0	2.07	73.00
	**Group J: unsaturated fatty acid**				
**1.**	**Linolenic acid**	C_18_H_30_O_2_ (278.0)	278.0	1.26	79.00
	**Group H: Esters**				
**1.**	**Bis (2-ethylhexyl) phthalate**	C_24_H_38_O_4_ (390.0)	390.0	7.73	149.0

## Data Availability

The original contributions presented in the study are included in the article, further inquiries can be directed to the corresponding author.
